# Estimation of Spatiotemporal Gait Parameters in Walking on a Photoelectric System: Validation on Healthy Children by Standard Gait Analysis

**DOI:** 10.3390/s23136059

**Published:** 2023-06-30

**Authors:** Silvia Campagnini, Guido Pasquini, Florian Schlechtriem, Giulia Fransvea, Laura Simoni, Filippo Gerli, Federica Magaldi, Giovanna Cristella, Robert Riener, Maria Chiara Carrozza, Andrea Mannini

**Affiliations:** 1 IRCCS Fondazione Don Carlo Gnocchi ONLUS, 50143 Firenze, Italy; scampagnini@dongnocchi.it (S.C.); gpasquini@dongnocchi.it (G.P.); fransveagiulia4@gmail.com (G.F.); laura.simoni@univr.it (L.S.); fgerli@dongnocchi.it (F.G.); fede_magaldi@hotmail.it (F.M.); amannini@dongnocchi.it (A.M.); 2 Department of Health Sciences and Technology, ETH Zurich, 8092 Zurich, Switzerland; schlflor@student.ethz.ch (F.S.); robert.riener@hest.ethz.ch (R.R.); 3 The BioRobotics Institute, Scuola Superiore Sant’Anna, 56025 Pisa, Italy; chiara.carrozza@santannapisa.it

**Keywords:** photoelectric system, gait analysis, kinematics, walking, child, rehabilitation

## Abstract

The use of stereophotogrammetry systems is challenging when targeting children’s gait analysis due to the time required and the need to keep physical markers in place. For this reason, marker-less photoelectric systems appear to be a solution for accurate and fast gait analysis in youth. The aim of this study is to validate a photoelectric system and its configurations (LED filter setting) on healthy children, comparing the kinematic gait parameters with those obtained from a three-dimensional stereophotogrammetry system. Twenty-seven healthy children were enrolled. Three LED filter settings for the OptoGait were compared to the BTS P6000. The analysis included the non-parametric 80% limits of agreement and the intraclass correlation coefficient (ICC). Additionally, normalised limits of agreement and bias (NLoAs and Nbias) were compared to the clinical experience of physical therapists (i.e., assuming an error lower than 5% is acceptable). ICCs showed excellent consistency for most of the parameters and filter settings; NLoAs varied between 1.39% and 12.62%. An inverse association between the number of LEDs for filter setting and the bias values was also observed. Observations confirm the validity of the OptoGait system for the evaluation of spatiotemporal gait parameters in children.

## 1. Introduction

A reliable assessment of gait patterns is essential for understanding the in-depth mechanisms of human functions, especially in clinical applications for performing diagnosis, monitoring gait deterioration, and keeping track of ambulation progress during rehabilitation programs [1,2]. Gait analysis allows for the quantification of human gait in the form of spatial, temporal, kinematic, and kinetic parameters such as walking speed, cadence, step length, stance, swing, and double support time [3].

Different reliable methods are currently available for the assessment of gait parameters, from more traditional quantitative and semi-subjective tests and analyses to wearable sensors, floor sensors, image-based techniques, and more complex multi-sensorial integrated systems [4]. In the last decades, the experience of clinicians and specialists has been more often supported by objective and quantitative data offered by the aforementioned methods. However, different characteristics differentiate these technologies and the types of applications they can be used for.

Among the solutions available in the literature, wearable sensor systems are available in a wide selection (gyroscopes, electromyography, accelerometers, among others); they are preferred for their applicability to a great variety of environments [5] and their reduced cost. However, their susceptibility to artefacts (interference from external factors, estimation drifts, or artefacts generated by the movement of tissues) makes them less accurate, and oftentimes complex algorithms are needed in order to extract parameters, especially in online applications [6,7].

On the other hand, GAL [8] is a commonly adopted reference system for gait analysis, allowing higher accuracy than the aforementioned methods [9]. The spatiotemporal gait parameters are obtained by measuring the kinematics and kinetics of the main body segments and joints: instantaneous positions of markers located on the participants’ skin surface are obtained through stereophotogrammetry, while external forces and contact times of the feet on the ground are measured by force plates. To ensure a natural walking pattern, force plates are often embedded into the floor and hidden from the patient [10,11]. Nevertheless, these marker-based solutions are also suffering from drawbacks. For instance, the acquisition of accurate measurements could be compromised by improper positioning or movement of the markers during the trial or by skin/soft tissue artefacts [12]. Moreover, they are organised as complex laboratories with expensive instrumentations and are therefore constrained by structured environments and size limitations, which make it impossible to collect more than four/five consecutive steps [4,13].

To overcome these drawbacks, according to the modern concept of ecological validity [14], Photocell Array devices (PA) have been introduced, allowing for the quantification of spatiotemporal gait parameters in less structured environments [15]. The OptoGait system (Microgate, Bolzano, Italy) is an example of these PA devices based on the transmission and reception of light between pairs of photoelectric cell bars. When participants walk between the bars, their feet pass through the light barriers, blocking the transmission between the photoelectric cells and therefore enabling the automatic detection of initial and end contact.

Thus, PA devices are suggested as new solutions with two main advantages. On one side, they allow us to overcome the costs and size constraints of the GAL; on the other side, they offer more robust data acquisition compared to wearable sensors [2,15]. Furthermore, PA devices could simplify the gait analysis process when working with children, easing both the identification of gait abnormalities and treatment planning [16]. Indeed, tasks such as prolonged standing for the calibration, wearing markers during the walkway, and having long data acquisition durations are significantly more difficult for children compared to adults [17,18]. Moreover, the ease of use of PA devices in unobstructed environments could improve the ecological validity of gait assessment, especially in children, who are normally easily distracted by instrumentation [3,19].

Previous research confirmed the validity of the spatiotemporal gait parameters obtained by the OptoGait system for healthy adults compared to high-speed video analysis [20], the GAITRite walkway [2], an instrumented treadmill [21], and a three-dimensional motion capture system [22]. The test-retest reliability of the OptoGait measurements in different sessions was also confirmed [2,15,21,23].

Despite its advantages and great exploitability, especially in paediatric applications, the PA solution has not yet been validated for paediatric gait assessment. There is no evidence that the available technical solution can be scaled to smaller anthropomorphic characteristics and different walking speeds/cadences in children. Indeed, in healthy children, gait parameters are dependent on age and height, and some changes can be observed during growth [18,24] until at least 14 to 16 years, with considerable changes, especially during the first 8 to 10 years [18,25]. The study of gait patterns at early stages can be important to investigate motion maturation and gait stabilisation and, therefore, to detect when pathology is influencing gait regularity earlier [26].

To summarise, PA systems represent a valid trade-off among the two most common tools available in the literature and clinical practice for gait analysis: GAL systems and wearable technologies. In fact, they can allow sufficient accuracy in gait parameter estimation as well as ease of use. These characteristics are particularly favourable in children’s gait analysis; however, validation of PA accuracy on smaller anthropomorphic measures is essential.

Thus, the aim of this study is the validation of a PA system as a method to determine the spatiotemporal gait parameters of healthy children. The gait parameters are validated by comparison with those obtained from a GAL system, specifically a stereophotogrammetry device with force plates. Additionally, different configurations for the PA system will be compared.

## 2. Materials and Methods

### 2.1. Participants

The participants were recruited between June 2019 and September 2022 using a convenience sample of healthy children aged between 6 and 14.

The exclusion criteria for the participants were:

- reported pain or injuries to the lower limbs within the previous six months,
- prior foot surgery,
- congenital or acquired foot deformities upon clinical examination,
- any disability that might affect the gait (e.g., flat feet, use of walking aids, visual or hearing impairment, or spine problems that might affect gait).

The study was approved by the Local Ethics Committee, “Comitato Etico Regionale per la Sperimentazione Clinica Regione Toscana–Sezione Comitato Etico Pediatrico” (Nr.41/2019), and before study participation, all participants signed informed consent.

### 2.2. Experimental Setting

A 6 m PA system (OptoGait, Microgate, Italy) and a stereophotogrammetry system with four force plates (BTS GAITLAB) were used in this study. The stereophotogrammetry system was set up to capture, through a 6-camera BTS motion capture system (BTS Smart DX, BTS Bioengineering, Garbagnate Milanese, Italy), sampling at 100 Hz, an 8 m long walkway with four force plates (AMTI OPT464508HF sampling at 1000 Hz; AMTI, USA) located at half of the distance. The PA system consisted of 6 pairs of bars (100 cm × 8 cm each). Each pair is constituted of a transmitting and receiving bar containing 96-port diodes (LEDs). In such a device, the diodes for detecting foot presence are located 3 mm above the floor level and approximately 1 cm apart. The PA system was placed approximately 40 cm from the starting point, in order to start the recording once a steady velocity is obtained.

Participants were asked to walk barefoot and to start at a self-selected velocity. Data were sampled at 1000 Hz and saved on a PC using OPTOGait Version 1.6.4.0 software (Microgate S.r.l., Bolzano, Italy).

Each participant was tagged with the relevant reflective markers according to the Davis protocol [27]. First, participants were asked to perform one familiarisation trial. After the familiarisation, they performed multiple experimental trials (with a minimum of three valid attempts), during which data from the OptoGait system and the BTS system were concomitantly collected.

Within every session, each walking trial was considered valid only if the participant stepped correctly on the force platforms, if the markers were visible and stable on the participant’s body, and if the participant performed an unaltered and uninterrupted walk.

### 2.3. Data Extraction

In the present study, heel strike was determined by BTS kinematic analysis and designated the beginning of each gait cycle, as in standard Gait Analysis.

OptoGait software (though GaitR IN and OUT filter options) allows us to adjust the minimum number of LEDs to be interrupted in order to properly identify a contact event. By changing this filter, it is possible to reduce or eliminate discrepancies between the OptoGait system and other gait analysis systems [19]. Given the existing literature and the company recommendations, the data were re-filtered by adjusting the OptoGait GaitR IN and OUT filter settings to 1 LED (i.e., the gait event is considered valid only when 1 additional LED is interrupted), 2 LEDs, or 3 LEDs.

Only steps correctly and simultaneously recorded by both systems were retained. Below, a list of the extracted spatiotemporal parameters extracted is presented:

Spatial parameters

- Step length (m), Anterior-posterior distance from the heel of one footprint to the heel of the opposite footprint;
- Stride length (m), Anterior-posterior distance between heels of two consecutive footprints of the same foot (left to left, right to right).

Temporal parameters

- Stance time (s), the time period between the initial contact and the consecutive end contact of the same foot;
- Swing time (s), the time period between the end contact and the consecutive initial contact of the same foot;
- Stride time (s), the time elapsed between the initial contacts of two consecutive footfalls of the same foot;
- Cadence (strides/min), the total number of full cycles taken within a given period of time.

Spatiotemporal parameters

- Velocity (m/s), the average speed of the gait cycle.

All the parameters, except for velocity and cadence, were extracted for each side separately, and they were quantified as the median of all steps from each participant.

### 2.4. Statistical Analysis

All statistical analyses were performed in MATLAB Version 9.13.0 (R2022b) Update 2. Descriptive analyses were performed, calculating the mean and standard deviation (std), median and interquartile range [IQR], and frequencies for continuous variables with normal distribution, continuous non-normally distributed variables, and categorical variables, respectively. The normality of the distribution was performed using the Shapiro–Wilk test, with a statistically significant *p*-value < 0.05.

Agreement between the OptoGait and the BTS system was examined using the Limits of Agreement (LoA) by Bland and Altman for the 12 previously described gait parameters (*p_j_*; *j* = 1 … 12) and the 3 filters (*f_i_*; *i* = 1, 2, 3) defined by the minimal number of LEDs that must be interrupted to detect a gait event.

Furthermore, the intraclass correlation coefficients (ICC) with the respective 95% confidence intervals were estimated in order to investigate the reliability. Based on the characteristics of the experimental design and following the guidelines reported by Koo et al. [28], the authors decided to conduct a “two-way random-effect model” with a “single rater” type and “consistency” definition for the ICC estimation (ICC(C, 1)) [29]. Values less than 0.5 were considered indicative of poor consistency, values between 0.5 and 0.75 for moderate consistency, values between 0.75 and 0.9 for good consistency, and values greater than 0.90 for excellent consistency [28].

Due to the reduced sample size, the Limits of Agreement were estimated non-parametrically, as suggested by Bland and Altman [30,31]. The upper and lower Limits of Agreement were estimated by, respectively, calculating the 10% and 90% quantiles of the paired differences’ distribution. In order to appropriately compare the accuracy between the three filter settings without considering the influence of a bias, we additionally introduced the normalised Limits of Agreement (NLoA) as the normalised intervals between the non-parametric upper and lower Limits of Agreements [32]. Both the bias and the NLoAs were calculated for each parameter $p_{j}$ and filter setting $f_{i}$ and were normalised with respect to the median of the measurements obtained from the optical motion capture as follows (Equations (1) and (2)).

(1) $$NLoA_{p_{j},f_{i}}=(\frac{LoA_{upper,\;p_{j},f_{i}}-LoA_{lower,\;p_{j},f_{i}}}{median\left(BTS_{p_{j}}\right)})*100\%$$

(2) $$NB_{p_{j},f_{i}}=(\frac{Bias_{p_{j},f_{i}}}{median\left(BTS_{p_{j}}\right)})*100\%$$

The NLoAs were evaluated through the clinical experience of physical therapists and classified with good (≤5%), poor (>10%) or moderate agreement (elsewhere).

Statistical analyses on outcome measures were conducted using MATLAB and Statistics Toolbox Release 2019b, The MathWorks, Inc., Natick, MA, USA.

## 3. Results

A total of 27 healthy children were included in the study. Participants’ characteristics and their kinematic parameters are shown in Table 1 and Table 2, respectively. The participants did not present any signs of cardiovascular, neurologic, or musculoskeletal disease.

**Figure 1:**
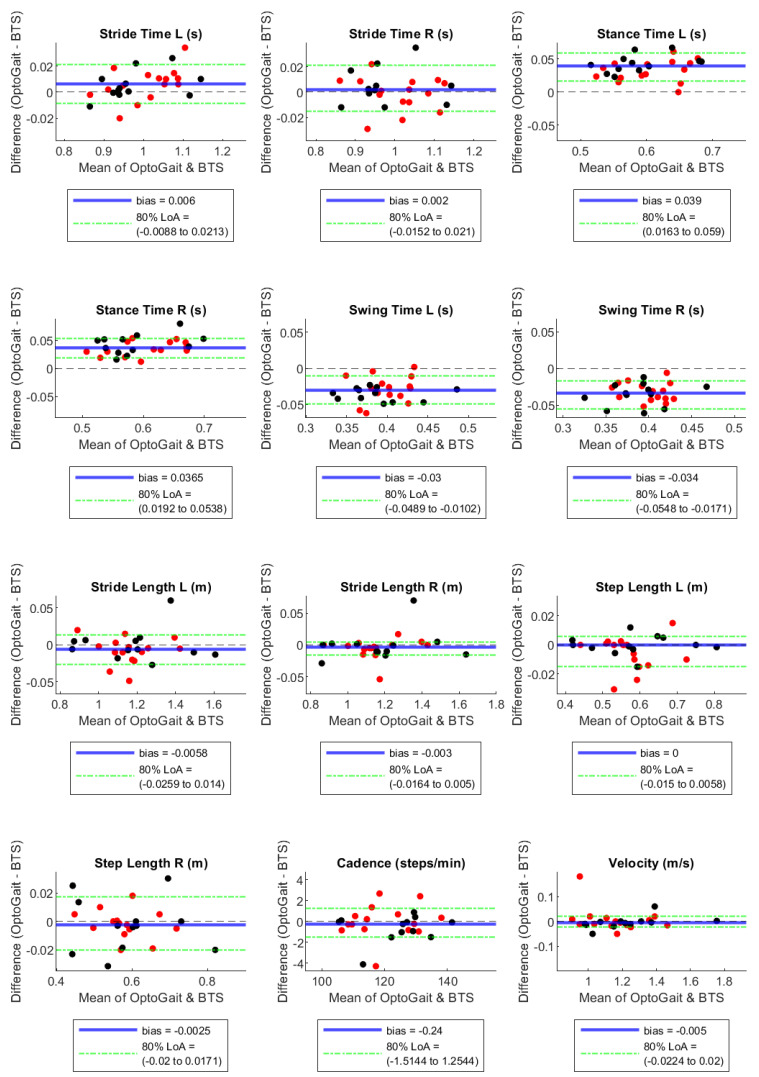
Bland–Altman plots with bias and 90% Limits of Agreement for filter-setting LED 1. Red and black dots are in correspondence with female and male participants, respectively.

**Figure 2:**
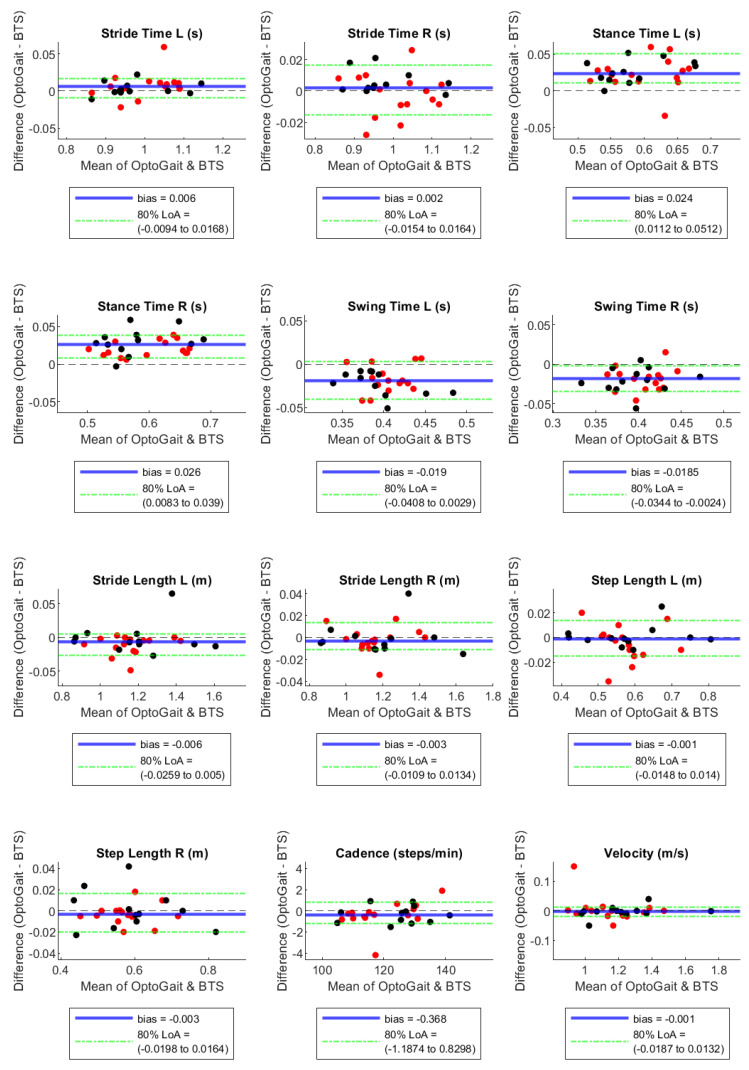
Bland–Altman plots with bias and 90% Limits of Agreement for filter-setting LED 2. Red and black dots are in correspondence with female and male participants, respectively.

**Figure 3:**
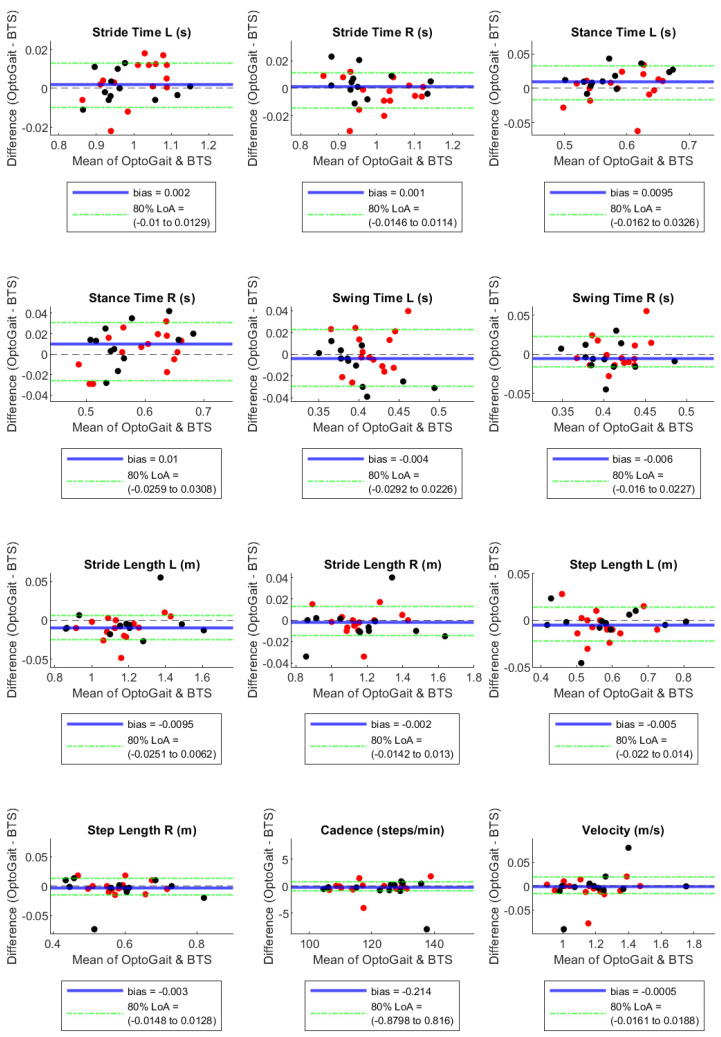
Bland–Altman plots with bias and 90% Limits of Agreement for filter-setting LED 3. Red and black dots are in correspondence with female and male participants, respectively.

By the Bland–Altman plots, Stance Time and Swing Time, both on the right and left sides, showed considerable bias with opposite signs, with absolute values of the normalised bias greater than 5% in configuration 1 LED, near 5% in configuration 2 LED, and much lower in configuration 3 LED (Table 3). In particular, Stance Time was overestimated and Swing Time was underestimated by the OptoGait system, with a decreasing tendency of these effects with the increase in the LED number for the filter setting (Table 3 and Figure 1, Figure 2 and Figure 3). For the remaining parameters, no considerable biases were observed.

As stated in the previous paragraph, from the Bland Altman LoAs, we calculated the NLoAs and compared them with the clinical standards provided by the physical therapists. The majority of parameters showed good NLoAs in the three configurations. However, as well as the bias, Swing and Stance Times revealed the biggest NLoA. In particular, the Swing Time L showed poor agreement in 3 LED and 2 LED filter settings, with an NLoA of 12.62% and 10.66%, respectively. The other configurations in Swing Times, Stance Times, and Step Lengths in most configurations showed moderate agreement (Table 4).

The estimated ICCs indicated excellent consistency across all gait parameters and filter settings, except for Swing Times, with 1 LED and 2 LED filter settings, and Swing Times with 1 LED and 2 LED configuration settings, reporting moderate and good consistency, respectively. Further, it is worth noting how all ICC reported comparable values, except for the aforementioned temporal parameters, where in particular the 1 LED setting is reporting a marked decrease (Table 5).

Comparing the different LED filter settings, 3 LED showed the lowest biases for 9 out of 12 parameters. The NLoAs were the smallest for 2 LED and 3 LED configurations, resulting in the best solutions for 5 and 4 parameters, respectively. In average terms, the 3 LED configuration has the lowest NBs, and the 2 LED ones show the lowest NLoAs. In terms of ICC, the three solutions show very similar average performances, with ICCS slightly decreasing while increasing the number of LEDs.

## 4. Discussion

The OptoGait system showed high concurrent validity with the motion capture system based on a comparison of spatiotemporal gait parameters of 27 healthy children obtained through 3 different filter settings (1 LED, 2 LED, and 3 LED). The results on the ICCs confirmed the consistency of the two measures, showing excellent values in all cases, with the exception of moderate values for Swing Times, with 1 LED and 2 LED filter settings and good values for Stance Times with 1 LED and 2 LED configuration settings. Moreover, the NLoAs showed good agreement, confirming the OptoGait system as a sufficiently reliable and accurate tool. The results of the bias obtained were further confirmed as acceptable after comparison with the findings reported by Oeffinger et al. [33] on a cohort of children with cerebral palsy. For this purpose, a 5% value for the bias was empirically selected based on the physiotherapists’ experience. Thus, the experience-based threshold was further confirmed by comparison with the Minimum Clinically Important Difference (MCID) values obtained by Hallman-Cooper et al. [34]. Indeed, the authors calculated the MCID values on cadence, stride length, and velocity and presented them as the minimum percentage of the parameters allowed for having a clinically important difference. For all the parameters, the retrieved MCID was always a percentage higher than 5% (cadence: 8.1%, stride length: 5.8%, velocity: 9.1%), indicating coherence of our experience-based acceptability limits with a diagnostic application for one of the main causes of disability in children’s populations.

It has to be noticed that we observed differences higher than 2% between the left and right NLoAs for the same parameter in two cases, specifically for the Swing Time (2 LED and 3 LED) and Step Length (1 LED). These differences could be explained by the reduced sample size, which enhances random effects such as those related to inaccurate marker placement.

A systematic bias higher than 1% between the motion capture and the PA system occurred for Stance and Swing Times in all configurations (Figure 1, Figure 2 and Figure 3). However, we observed a bias reduction in temporal parameters by increasing the number of LEDs in the filter setting. Several studies have already reported a longer stance time and a shorter swing time recorded by PA compared to other instruments such as motion capture [22], walkway [2,3], and treadmill [21] systems. The temporal gap is explained by the fact that the OptoGait photoelectric diodes are raised 3 mm above the ground. Hence, it starts measuring stance time before heel strike, when the foot is detected by the diodes but is not in contact with the floor yet. The force sensors, instead, are able to identify the exact moment when the heel strikes the ground due to the high sensibility of the platform (default triggered at 30 N). For the same reason, OptoGait starts measuring toe-off later than the motion capture system, and this causes an underestimated OptoGait swing time. This aspect is in agreement with previous literature on the validation of PA devices on adults’ gait [22].

By adding LEDs to the filter settings, the interruption of additional LEDs is required for heel-strike and toe-off detection, allowing the user to configure the setting that reduces most of the discrepancies with the other measurement system.

Our results showed the lowest values of bias with the 3 LED configuration. The filter configurations with 2 LEDs showed slightly better performances in terms of NLoAs than other configurations. More specifically, the 2 LED showed better performances on the five parameters (Stride Length R, Swing Time R, Velocity, and Stance Times), whereas for the four parameters (Step Length R, Stride Times, and Cadence), the 3 LED setting obtained the best results. The 2 LED configuration never showed the worst result among the three configurations in terms of NLoAs. Moreover, considering the parameters for which the 2 LED setting was not preferred, the average difference in NloAs between 2 LED and the preferred configuration was below 1.5%. Concerning ICC, the 3 LED configuration shows the best result for 8 out of 12 parameters, with an average value of ICCs that is very similar across configurations, with a slight decrease for the 2 LED and a marked decrease in the 1 LED in the temporal parameters of Swing and Stance Times.

Based on these considerations of NLoAs and biases, we would recommend the use of the 2 LED filter setting for the measurement of healthy children’s kinematic gait parameters with the OptoGait system. This result is in agreement with the findings of other studies carried out on healthy adults, which recommended the use of 1 LED or 2 LED configurations when compared to high-speed video analysis [20] and stereophotogrammetry [22], respectively. Even if these results were derived from a relatively small sample size, the selection of a higher filtering configuration (2 LED) could be more likely explained by the weight difference between adults and children, as well as different experimental configurations (treadmill and overground walking, high-speed video analysis, and stereophotogrammetry). The heel strike event is recorded by the force platform earlier in an adult than in a child due to the sensitivity limit of the platform. Consequently, the stance time measured on children using force platforms tends to be reduced compared to adults. Thus, higher filter settings are needed for children’s applications than adults’, since the aim of the LED filtering is to introduce delay for an accurate stance time measurement. Similarly, the same concept applies to the toe-off event, which is recorded later in an adult than in a child. Consequently, the swing time measured by the force platform in a child is higher than in an adult.

To conclude, the results obtained confirmed the validity of the OptoGait device for the evaluation of gait in children. Being non-invasive and easy to use, this device has great potential for integrating the analysis of gait in ecological settings. Specifically, the gait analysis of children is essential to further promote research investigating the processes of motion maturation and the early detection of impairments [26].

## 5. Conclusions

Our findings confirm the validity of the OptoGait system for the evaluation of spatiotemporal gait parameters in healthy children, as it has already been reported in the literature for healthy adults. However, additional research is required to confirm its validity with a larger sample size, which promotes the use of parametric statistical tools and allows for more robust recommendations in terms of filter settings. Moreover, instead of using a unique filtering setting for the GaitR IN and OUT phases of the PA detection, different combinations could be investigated to obtain a more appropriate tuning for children to achieve better performances.

Further developments should consider larger cohorts and inter-operator variabilities, as well as analysis of different populations, such as participants with pathological conditions, and comparisons of different gait modalities, such as running or multiple walking speeds.

## Figures and Tables

**Table 1:** Participants’ characteristics (12 males and 15 females).

	Descriptive Statistics (Median [Interquartile Range])
Age (years)	9 [4]
Height (cm)	133 [27]
Weight (kg)	32 [20]

**Table 2:** Participants’ gait parameters.

Parameters (Units)	Descriptive Statistics (Median [Interquartile Range])
Stride Time L (s)	0.970 [0.122]
Stride Time R (s)	0.965 [0.107]
Stance Time L (s)	0.580 [0.094]
Stance Time R (s)	0.562 [0.085]
Swing Time L (s)	0.405 [0.046]
Swing Time R (s)	0.420 [0.045]
Cadence (steps/min)	124.0 [15.53]
Stride Length L (m)	1.184 [0.164]
Stride Length R (m)	1.157 [0.179]
Step Length L (m)	0.571 [0.092]
Step Length R (m)	0.584 [0.062]
Velocity (m/s)	1.190 [0.255]

**Table 3:** Normalised Bias, i.e., bias as a percentage of the median obtained from BTS.

	$NB_{p_{j},f_{i}}$		
	1 LED	2 LED	3 LED
Stride Time L	0.619	0.619	**0.206**
Stride Time R	0.207	0.207	**0.104**
Stance Time L	6.724	4.196	**1.661**
Stance Time R	6.495	4.626	**1.779**
Swing Time L	−7.407	−4.691	**−0.988**
Swing Time R	−8.095	−4.405	**−1.429**
Cadence	−0.194	−0.297	**−0.173**
Stride Length L	**−0.490**	−0.507	−0.803
Stride Length R	−0.259	−0.259	**−0.173**
Step Length L	**0.000**	−0.175	−0.876
Step Length R	**−0.428**	−0.514	−0.514
Velocity	−0.420	−0.084	**−0.042**
*Mean absolute NB*	*2.612*	*1.715*	***0.729***

Note: in bold are indicated the LED configurations with lower bias percentages.

**Table 4:** Normalised limits of agreement as a percentage of the median obtained from BTS.

	$NLoA_{p_{f}f_{t}}\;\left[\%\right]$		
	1 LED	2 LED	3 LED
Stride Time L	3.04	2.64	**2.31**
Stride Time R	3.64	3.20	**2.62**
Stance Time L	7.35 *	6.90 *	8.41 *
Stance Time R	5.97 *	5.30 *	9.80 *
Swing Time L	9.43 *	10.66 **	12.62 **
Swing Time R	9.17 *	7.78 *	9.40 *
Cadence	2.27	1.65	**1.39**
Stride Length L	3.40	**2.63**	2.66
Stride Length R	**1.84**	2.08	2.33
Step Length L	**3.59**	4.97	6.21 *
Step Length R	6.35 *	6.19 *	**4.72**
Velocity	3.58	**2.69**	2.94
*Mean NLoA*	*4.97*	***4.72***	5.45 *

Note: * is indicating a moderate agreement (5% < NLoA ≤ 10%); ** is indicating a poor agreement (NLoA > 10%); in bold are represented the LED configurations with greater measurement agreement.

**Table 5:** Intraclass correlation coefficient (ICC) and 95% Confidence Interval (CI) between measurements from BTS and OptoGait.

	1 LED			2 LED			3 LED		
	ICC	95% CI		ICC	95% CI		ICC	95% CI	
		Lower Bound	Upper Bound		Lower Bound	Upper Bound		Lower Bound	Upper Bound
Stride Time L	0.986	0.967	0.995	0.982	0.958	0.992	**0.993**	0.984	0.997
Stride Time R	0.987	0.971	0.994	0.990	0.979	0.995	**0.991**	0.980	0.996
Stance Time L	0.765	−0.055	0.941	0.841	0.083	0.955	**0.916**	0.823	0.961
Stance Time R	0.783	−0.048	0.948	0.884	0.013	0.972	**0.943**	0.876	0.974
Swing Time L	0.648	−0.075	0.898	0.772	0.016	0.930	**0.850**	0.701	0.929
Swing Time R	0.563	−0.066	0.865	0.735	−0.013	0.917	**0.816**	0.636	0.912
Cadence	0.989	0.977	0.995	**0.994**	0.987	0.997	0.984	0.965	0.993
Stride Length L	0.994	0.987	0.997	0.994	0.986	0.997	**0.995**	0.987	0.998
Stride Length R	0.994	0.988	0.997	**0.998**	0.995	0.999	0.997	0.994	0.999
Step Length L	**0.994**	0.986	0.997	0.991	0.981	0.996	0.986	0.970	0.994
Step Length R	**0.988**	0.974	0.994	**0.988**	0.974	0.995	0.983	0.963	0.992
Velocity	0.978	0.952	0.990	0.984	0.966	0.993	**0.988**	0.975	0.995
*Mean ICC*	*0.889*	*0.630*	*0.968*	*0.929*	*0.660*	*0.978*	***0.953***	*0.904*	*0.978*

Note: in bold are indicated the LED configurations with higher ICC.

## Data Availability

Data will be provided upon request to the corresponding author for research purposes.
